# Cost-effectiveness of cervical cancer screening and preventative cryotherapy at an HIV treatment clinic in Kenya

**DOI:** 10.1186/s12962-017-0075-6

**Published:** 2017-07-14

**Authors:** Marita R. Zimmermann, Elisabeth Vodicka, Joseph B. Babigumira, Timothy Okech, Nelly Mugo, Samah Sakr, Louis P. Garrison, Michael H. Chung

**Affiliations:** 1 Department of Pharmacy, University of Washington, 1959 NE Pacific St., HSB H-375, Box 357630, Seattle, WA 98195 USA; 2 Department of Global Health, University of Washington, 325 Ninth Avenue, Box 359909, Seattle, WA 98104 USA; 3 Chandaria School of Business, United States International University-Africa, Nairobi, Kenya; 4 Department of Obstetrics and Gynecology, Kenyatta National Hospital, Nairobi, Kenya; 5 Coptic Hospital, Coptic Hope Center, Nairobi, Kenya; 6 Department of Medicine, University of Washington, Seattle, WA USA; 7 Department of Epidemiology, University of Washington, Seattle, WA USA

**Keywords:** Cervical cancer, Cost effectiveness, HIV, VIA, Cryotherapy

## Abstract

**Objective:**

This study evaluated the potential cost-effectiveness of cervical cancer screening in HIV treatment clinics in Nairobi, Kenya.

**Methods:**

A Markov model was used to project health outcomes and costs of cervical cancer screening and cryotherapy at an HIV clinic in Kenya using cryotherapy without screening, visual inspection with acetic acid (VIA), Papanicolaou smear (Pap), and testing for human papillomavirus (HPV). Direct and indirect medical and non-medical costs were examined from societal and clinic perspectives.

**Results:**

Costs of cryotherapy, VIA, Pap, and HPV for women with CD4 200–500 cells/mL were $99, $196, $219, and $223 from a societal perspective and $19, $94, $124, and $113 from a clinic perspective, with 17.3, 17.1, 17.1, and 17.1 years of life expectancy, respectively. Women at higher CD4 counts (>500 cells/mL) given cryotherapy VIA, Pap, and HPV resulted in better life expectancies (19.9+ years) and lower cost (societal: $49, $99, $115, and $102; clinic: $13, $51, $71, and $56). VIA was less expensive than HPV unless HPV screening could be reduced to a single visit.

**Conclusions:**

Preventative cryotherapy was the least expensive strategy and resulted in highest projected life expectancy, while VIA was most cost-effective unless HPV could be reduced to a single visit.

## Background

Cancer is a growing cause of mortality worldwide, and cervical cancer is one of the leading causes of cancer death in sub-Saharan Africa (SSA) [1]. Although preventable if detected early, cervical cancer is one of the most prevalent cancers on the continent with 75,000 incident cases per year [2, 3]. In addition to the cancer burden, more than 10 million women are infected with HIV and are therefore at greater risk for cervical cancer and early mortality, making early detection and prevention critical for this already vulnerable population [4, 5].

Reducing cervical cancer among HIV-infected women is a primary focus of the Pink Ribbon Red Ribbon Initiative, a joint public–private international program launched in 2011 supported by the President’s Emergency Plan for AIDS Relief (PEPFAR) [6]. The initiative promotes integrating cervical cancer screening and treatment into HIV treatment clinics in sub-Saharan Africa [6]. Understanding the cost-effectiveness of each cervical cancer screening method in an integrated context is essential to meeting the goals of the Initiative and to sustaining cervical cancer screening programs during a period of decreasing PEPFAR funding [6].

Our recent cross-sectional study at an HIV treatment clinic in Kenya in which 498 women all received Pap, VIA, and HPV screening, found human papilloma virus testing with a Cervex brush in PreservCyt media (HPV) to be the most sensitive cervical cancer screening method among HIV-infected women (81%) followed by Pap smear (74%) and visual inspection with acetic acid (VIA) (61%), while Pap smear was the most specific (98%) followed by VIA (63%) and HPV (55%) [7]. However, VIA screening and treatment has been shown to be feasible, acceptable, and effective in detecting and treating pre-cancerous cervical lesions [8–16]. In Kenya, VIA, VIA/VILI, Pap, and HPV are all recommended for screening in Kenya [17], though the World Health Organization (WHO) recommends human papillomavirus (HPV)-based screening tests in countries that have not established an effective, high-coverage Pap-based program [18].

Furthermore, a recent abstract suggested that preventative cryotherapy for women of screening age may yield greater health benefits than once-in-a-lifetime screening [19]. Cryotherapy has been shown to be effective and have relatively low risks [20, 21]. Particularly in settings where access to screening or cancer treatment may be limited, preventative cryotherapy may be an effective and affordable alternative.

In this study we compared the cost-effectiveness of Pap, VIA, and HPV screening, combinations of these screens, and preventative cryotherapy in an HIV clinic using primary cost data and screening test sensitivity and specificity based on the gold standard of colposcopy-directed biopsy. We modeled the costs and effectiveness of each cervical cancer screening strategy over the lifetime of an average HIV-infected patient to estimate which methods might be provide the best health outcomes while minimizing costs.

## Methods

### Study site and population

The Coptic Hope Center for Infectious Diseases in Nairobi, Kenya, is an HIV comprehensive care clinic where HIV-infected men, women, and children receive antiretroviral therapy (ART) and treatment for opportunistic infections [22]. The Hope Center was established in 2004 by the University of Washington and the Coptic Christian Mission with funding from PEPFAR through the Centers for Disease Control and Prevention (CDC). Cervical cancer screening has been offered at the Hope Center since 2005 with over 7000 HIV-infected women having been screened to date [22].

In 2009, we conducted a cross-sectional study of cervical cancer screening with 498 HIV-infected women at the Hope Center to compare VIA, Pap smear, and HPV (Cervex brush with PreservCyt media) against the gold colposcopy-directed biopsy [7]. Sensitivity, specificity, and overall accuracy of each screening method (defined by area under the receiver operator curve) were compared using pairwise tests and multivariable logistic regression models that included age, CD4 count, and ART duration. The cost analysis presented here was based on resource use for each method derived from the Coptic Hope Center.

### Natural history model

**Fig. 1:**
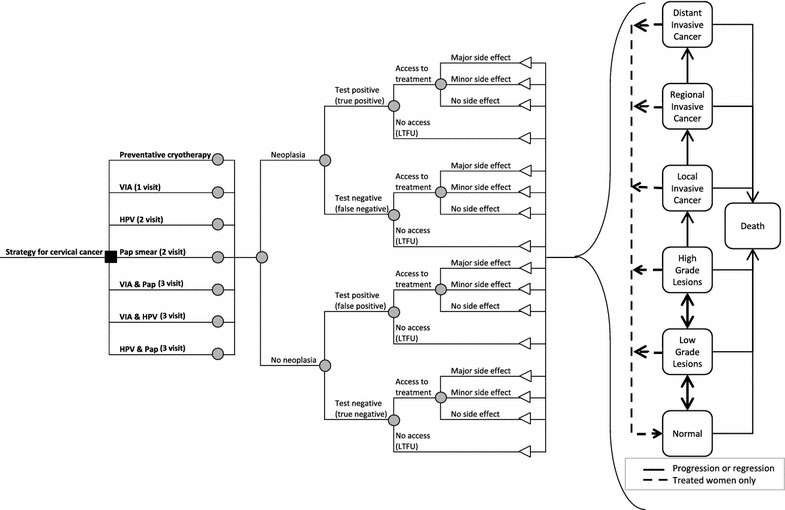
Decision tree representing pre-cancerous lesions, cancer, testing, treatment, and side effects follow by summary states in Markov model. *Pap* Papanicolaou smear, *HPV* DNA testing for Human Papilloma Virus, *VIA* visual inspection of the cervix with acetic acid

A Markov model was used to simulate the natural history of cervical cancer in HIV-infected women based on a framework by Goldie et al. [23]. that has been used extensively to model cervical cancer among HIV-infected patients [24–26]. In the model, the health states represented HPV DNA status, the grade of lesion, and the stage of invasive cancer (Fig. 1). Movement through the health states occurred in monthly increments according to probabilities (Table 1). Costs and benefits were discounted at an annual rate of 3% (0.25% monthly), consistent with economic guidelines [27]. Modeling was completed using TreeAge Pro 2013 [28].

**Table 1:** Base case parameters and sensitivity analysis parameters used in modeling cervical cancer in HIV-infected women

	Base case		One-way sensitivity analysis	CD4 scenario sensitivity analyses		Source
	CD4 200–500		Min–max	CD4 ≤ 200	CD4 > 500	
Start age	38		30–46	N/A	N/A	[7]
Initial prevalence of cancer and pre-cancerous lesions	0.22		0.18–0.27	0.33	0.12	[7]
Initial prevalence						
Among cancer/pre-cancerous lesions						
Low grade lesions	0		N/A	N/A	N/A	[12, 23]
High grade lesions	0.952		0.762–1	N/A	1	
Local cancer	0.020		0.016–0.024	N/A	0	
Regional cancer	0.024		0.019–0.029	N/A	0	
Distant cancer	0.004		0.003–0.005	N/A	0	
Among normal						
Low grade lesions	0.098		0.078–0.118	0.169	0.025	[23]
Normal	0.902		0.722–1	0.832	0.975	
Loss-to-follow-up probability						
2-visit	0.32		0–0.4	N/A	N/A	[29, 52]
3-visit	0.48		0.3–0.6	N/A	N/A	[29, 52]
Standardized mortality ratio for treated HIV-infected patients compared to HIV negative	1.77		1.42–2.12	2.54	1	[53]
Untreated cancer parameter						
Cancer mortality probability (monthly)						
Local	0.004		0.003–0.005	N/A	N/A	[24]
Regional	0.021		0.017–0.025	N/A	N/A	
Distant	0.063		0.051–0.076	N/A	N/A	
Progression probability (monthly)						
Normal to low grade lesions	0.001		0.000–0.002	N/A	0.000	[23, 24]
Low grade to high grade lesions	0.003		0.002–0.004	N/A	0.001	
High grade lesions to local invasive cancer	0.002		0.001–0.003	N/A	0.002	
Local to regional invasive cancer	0.003		0.002–0.004	N/A	N/A	
Regional to distant invasive cancer	0.003		0.002–0.004	N/A	N/A	
Regression probability (monthly)						
Low grade lesions to normal	0.003		0.002–0.004	N/A	N/A	[24]
High grade to low grade lesions	0.000		0.000–0.001	N/A	N/A	
High grade lesions to normal	0.000		0.000–0.001	N/A	N/A	
Treated cancer parameters						
Treatment effectiveness (mortality, progression, lesion regression)	0.9		0.7–1	N/A	N/A	[11]
Treated cancer cure probability (monthly)	0.15		0.12–0.18	N/A	N/A	[12]
Side effect probability (monthly)	0.01		0.00–0.02	N/A	N/A	[13]
Probability side effect is Major	0.16		0.13–0.20	N/A	N/A	
Sensitivity						[7]
Pap	0.74		0.59–0.89	0.76	0.61	
VIA	0.61		0.49–0.73	0.76	0.52	
HPV	0.81		0.65–0.97	0.92	0.83	
VIA + HPV	0.58		0.46–0.70	N/A	N/A	
VIA + Pap	0.51		0.41–0.61	N/A	N/A	
HPV + Pap	0.63		0.50–0.76	N/A	N/A	
Specificity						[7]
Pap	0.98		0.78–1	0.93	0.98	
VIA	0.63		0.50–0.76	0.62	0.73	
HPV	0.55		0.44–0.66	0.46	0.62	
VIA + HPV	0.84		0.67–1	N/A	N/A	
VIA + Pap	0.99		0.79–1	N/A	N/A	
HPV + Pap	0.99		0.79–1	N/A	N/A	

Costs (2014 USD)	Societal perspective	Clinic perspective				
Screening						[31]
PAP	$39	$24	$31–$47	N/A	N/A	
VIA	$18	$10	$14–$22	N/A	N/A	
HPV (CareHPV)	$32	$18	$26–$38	N/A	N/A	
Treatment						
Cryotherapy	$48	$12	$38–$58	N/A	N/A	
Colposcopy	$160	$109	$128–$192	N/A	N/A	
LEEP	$86	$20	$69–$103	N/A	N/A	
Side effects						
Major	$974	$847	$779–$1169	N/A	N/A	
Minor	$203	$178	$162–$244	N/A	N/A	
Cancer care						
Local	$1135	$112	$908–$1362	N/A	N/A	
Regional	$6447	$149	$5158–$7736	N/A	N/A	
Distant	$5107	$144	$4086–$6128	N/A	N/A	
Palliative care	$196	$145	$157–$235	N/A	N/A	

*min* minimum, *max* maximum, *Pap* Papanicolaou smear, *HPV* DNA testing for Human Papilloma Virus, *VIA* visual inspection of the cervix with acetic acid, *LEEP* loop electrosurgical excision procedure, *N/A* not applicable

### Model and strategies

Clinical strategies included: Pap smear, VIA, HPV, Pap plus VIA, Pap plus HPV, VIA plus Pap, and preventative cryotherapy with no screen (Fig. 1). For strategies including an HPV screen, women were considered to have a positive diagnosis if they tested positive for high-risk HPV types 16, 18, 31, 33, 35, 39, 45, 51, 52, 56, 58, 59, 66 or 68. For strategies including a Pap smear, women were considered to have a positive diagnosis if they had high-grade squamous intra-epithelial lesions or greater (HSIL+). True test positivity was defined as having CIN 2/3 or greater by colposcopy-directed biopsy. Each woman was assumed to receive a single screening for cervical cancer during her lifetime at 38 years of age, which was the mean age of women in the screening study.

Test performance (sensitivity and specificity) data was based on our recent cross-sectional study at an HIV treatment clinic in Kenya (Table 1) [7]. The median age of the population was 38 years and 57% were under age 50 [7]. Nearly half (43%) of the participants were married, and 51% had at least a secondary school education. Most women (77%) were employed, none reported any smoking history, and 25% reported having 3 or more lifetime sexual partners [7]. The median CD4 count at the time of cervical cancer screening was 371 cells/mL, with 16% having a low CD4 count (<200 cells/mL), and 28% having a high CD4 count (>500 cells/mL) [7]. Three hundred and seventy-seven women (75%) were on ART at the time of cervical cancer screening, and 182 (48%) of these women had been on ART for >2 years [7]. Those on ART had been taking antiretroviral medications for a median duration of 797 days (IQR 330–1210) [7].

For this model, women could receive screening and treatment in one, two or three visits. In a single-visit strategy, VIA screen and treatment with cryotherapy or LEEP were modeled to occur on the same day. Two-visit strategies consisted of an HPV or Pap screen, followed by provision of results and treatment (as needed) during a follow-up visit. Three-visit strategies included an initial screening visit, a second confirmatory screening visit for all women with positive results, and a treatment visit for all women with two positive results. We assumed a base case of loss to follow-up (LTFU) of 32% based on studied LTFU rates for cervical cancer screening in an ART clinic in Kenya [29]. Treatment options included cryotherapy, loop electrosurgical excision procedure (LEEP), hysterectomy, chemotherapy, radiotherapy, and palliative care (Table 2). For treatment for high-grade lesions, 80% of women received cryotherapy and 20% received LEEP.

**Table 2:** Base case treatment and costs included for each health state in the first month and subsequent months in that state for all cervical cancer screening strategies

Health state	Health services and costs included in first month			Health services and costs included in subsequent months
	Screening	Treatment	Treatment of any side effects	
No lesion	Yes	–	–	None
Low grade lesions	Yes	–	–	None
High grade lesions	Yes	Cryotherapy or LEEP	Yes	None
Local invasive cancer	Yes	Radical hysterectomy	Yes	None
Regional invasive cancer	Yes	Radical hysterectomy, Radiotherapy + chemotherapy	Yes	Palliative care
Distant invasive cancer	Yes	Radiotherapy + chemotherapy	Yes	Palliative care

*Pap* Papanicolaou smear, *HPV* DNA testing for Human Papilloma Virus, *VIA* visual inspection of the cervix with acetic acid, *LEEP* loop electrosurgical excision procedure

### Clinical data

Table 1 shows selected variables based on published literature. Primary data from Coptic Hope Center was used whenever possible. We used literature values from Kenya, SSA, or low-income settings to supplement when primary data was unavailable. The base case model represented women with CD4 count 200–500 cells/mL. Progression, regression, and mortality rates were not dependent on a previous history of lesions or cancer, therefore, potential for cancer recurrence was not included (parameters were aggregate representations of populations that included women with and without a history of lesions and cancer). Mortality rates were age-specific and sensitivity and specificity of screening was binary for age <40 and ≥40 at time of screen. Background mortality rates for the population were WHO age-specific all-cause mortality rates for Kenyan females in 2011 [30].

### Cost data

A quantity-and-price approach was used to estimate costs, based on primary data collected through a micro-costing study at Coptic Hope Center for Infectious Diseases and Kenyatta National Hospital, Kenya, between July 1 and October 31, 2014 [31]. Direct medical, non-medical, and indirect costs were estimated using a time-and-motion study ad semi-structured interviews with patients and clinic staff. This study found VIA to cost $3.30, careHPV to cost $18.28, and pap Pap to cost $24.59 per screen [31]. Indirect costs were lower for single-visit screening methods ($0.43 per screening) than two-visit screening methods ($2.88 per screening) [31]. Costs are presented in 2014 US dollars, the year of the costing study. The study team at Coptic Hope Center procures their supplies from local vendors and international supply distributors. As an HIV-treatment center, costs at Coptic Hope Center may differ from primary care and non-specialized public health facilities. However, our costs estimates were reasonably comparable to estimates from other HIV-treatment and public health facilities previously reported for Kenya and SSA [31].

Costs were categorized as direct medical costs (e.g. staff time, medical supplies), direct non-medical costs (e.g. overhead, patient transportation costs), and indirect costs (e.g. patient time, child care). The analysis was completed using two perspectives for cost. First, a societal perspective included direct medical, direct non-medical, and indirect costs to the health center, patient, and caregiver. This perspective accounts for patient out-of-pocket costs and the economic opportunity costs (e.g., missed wages) associated with a patient or caregiver’s time lost while receiving care. Second, a clinic perspective included direct medical and direct non-medical costs for the health care center only.

Costs included for each health state are shown in Table 2, delineated separately for the first month of treatment and subsequent months of treatment. For individuals with local, regional or distant invasive cancer, 3 round-trip visits entailed: (1) examination under anesthesia for staging and diagnosis, (2) minor theater day/first treatment, and (3) one return visit for histology report and/or development of management plan. HPV screening costs were modeled on the projected use of careHPV [32]. Cost of preventative cryotherapy included patient out-of-pocket costs, personnel, supplies, patient transport, non-medical costs, overhead, and lost wages. Costs included expenses resulting from false positive diagnoses and treatment complications.

## Results

Preventative cryotherapy was projected to have the lowest mean lifetime cost of screening and treatment ($99, societal perspective; $19, clinic perspective), as well as lead to the highest projected life expectancy (17.3 years from time of screening to death) (Table 3). VIA, Pap screening and HPV screening had higher projected mean lifetime costs of screening and treatment ($196, $219, $223, societal perspective; $94, $124, $113, clinic perspective, respectively), and lower life expectancy (17.1 years). The combination strategies of VIA + HPV, HPV + Pap, and VIA + Pap had higher mean lifetime costs of screening and treatment ($258, $261, and $263, societal perspective; $150, $155, and $158, perspective, respectively) and lower life expectancy (17.0 years).

**Table 3:** Lifetime costs and life expectancy results for base case and sensitivity analyses

Scenario	Strategy	Lifetime costs		Life expectancy (years from screening)
		Societal perspective	Clinic perspective	
Base case	Preventative cryotherapy	$99	$19	17.3
	VIA	$196	$94	17.1
	PAP	$219	$124	17.1
	HPV	$223	$113	17.1
	VIA + HPV	$258	$150	17.0
	HPV + PAP	$261	$155	17.0
	VIA + PAP	$263	$158	17.0
Sensitivity analyses				
Low CD4 (CD4 ≤ 200 cells/mL)	Preventative cryotherapy	$122	$22	15.3
	VIA	$208	$87	15.2
	HPV	$262	$125	15.2
	PAP	$281	$152	15.1
	VIA + HPV	$339	$194	15.0
	HPV + PAP	$342	$199	15.0
	VIA + PAP	$348	$205	15.0
High CD4 (CD4 > 500 cells/mL)	Preventative cryotherapy	$49	$13	20.0
	VIA	$99	$51	19.9
	HPV	$102	$56	19.9
	VIA + HPV	$105	$69	19.9
	PAP	$115	$71	19.9
	HPV + PAP	$119	$80	19.9
	VIA + PAP	$105	$72	19.8

*Pap* Papanicolaou smear, *HPV* DNA testing for Human Papilloma Virus, *VIA* visual inspection of the cervix with acetic acid

When the number of visits was reduced from two to one for Pap and for HPV in scenario analyses, the projected mean lifetime costs of screening and treatment decreased (from $219 to $168 for Pap and from $223 to $169 for HPV, societal perspective). Additionally, the years of life expectancy for Pap remained the same (17.1 years), but increased for HPV (from 17.1 to 17.2 years).

All treatment strategies among women with a CD4 count ≤200 cells/mL at time of screening yielded projected years of life that were approximately two years lower than women who had CD4 count 200–500 cells/mL. Among women with CD4 count ≤200 cells/mL, preventative cryotherapy remained the least expensive ($122, societal perspective; $22, clinic perspective) and had the highest life expectancy (15.3 years) (Table 3). Among women with CD4 count >500 cells/mL at time of screening, all treatment strategies increased projected life expectancies by approximately 2 years but decreased costs compared to women with CD4 200–500 cells/mL. Preventative cryotherapy remained the least expensive strategy ($49, societal perspective; $13, clinic perspective) with the highest projected life expectancy (20.0 years) (Table 3).

**Fig. 2:**
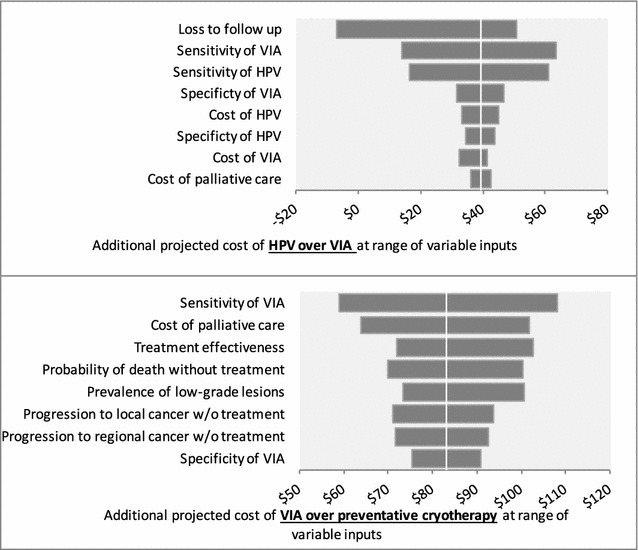
Tornado diagrams for sensitivity analysis of estimated additional costs of VIA over preventative cryotherapy (*top*) and HPV over VIA (*bottom*) at a range of input variables (see Table 1 for ranges). *VIA* visual inspection of the cervix with acetic acid, *Pap* Papanicolaou smear, *HPV* DNA testing for Human Papilloma Virus

Preventative cryotherapy was less expensive than VIA in all one-way sensitivity analyses, and VIA was less expensive than HPV in almost all analyses (Fig. 2). Holding all other variables constant, HPV was projected to have the same cost as VIA when LTFU was lowered from the base case (32%) to 17%.

## Discussion

Preventative cryotherapy without screening was projected to be the least expensive strategy for preventing cervical cancer in HIV-infected women and led to the highest projected life expectancy compared to VIA, Pap, HPV, and their combinations. Of the screening strategies, VIA was the least expensive and led to the highest projected life expectancy. If HPV could be reduced to a 1-visit strategy, it would be projected to be less expensive than VIA. For all screening strategies, was least costly and most effective to intervene on women with any test at CD4 counts >500 cells/mL.

Our results supported previous work that found preventative cryotherapy to be a cost-effective intervention in LMICs [19]. Given the high rate of test positivity in our HIV-infected population, the expense of screening, and the increased potential for LTFU in this resource-limited setting, it appears less expensive and more effective to treat all women with cryotherapy preventively. Prophylactic cryotherapy may also prevent acquisition of HPV that can lead to cervical cancer among those who test negative [33]. Cryotherapy may result in minor adverse events such as vaginal discharge, abdominal/lower back pain, or vaginal bleeding [34]. However, since cryotherapy appears safe to use without severe adverse effects related to pregnancy and without necessarily causing increased shedding of HIV virus from the cervix [35–37], it merits further investigation as a treatment tool that could help all women enrolled in an HIV clinic.

When comparing traditional screening methods, our results were consistent with other studies in LMICs that found VIA to be a cost-effective screening method for cervical cancer [11–16, 38]. Several studies have demonstrated that integration of VIA into routine HIV care in Africa is feasible and acceptable [39–42]. The results presented here further support integration of cervical cancer screening and HIV care. An overarching goal of the WHO global health sector strategy on HIV/AIDS is to achieve universal access to comprehensive HIV prevention, treatment, and care, including strengthening linkages between HIV and other related health programs, such as cervical cancer screening and treatment [43]. Demonstrating that cervical cancer screening or preventative treatment is a cost-effective strategy in this setting is an important step towards successfully reaching that goal. With combined efforts of the research community and organizations, such as the Pink Ribbon Red Ribbon campaign, integration into HIV settings would allow for many more women with cervical cancer to receive screening and treatment.

In contrast to our findings, some evaluations of non-integrated settings found HPV screening to be a more cost-effective screening method than VIA [44, 45]. Our model used primary, current data of costs and test sensitivity when providing screening in an integrated setting. Our cost estimates included direct and indirect costs over the lifetime of the patient, whereas other studies used narrower definitions of costs and time horizons [44, 45]. These factors likely led to the difference in conclusion from previous studies. Other studies also may differ due to variations in the assumptions made related to number of visits and timing of treatment, which have been shown to be of key importance regardless of screening type [13, 46–48]. Finally, a SIL-based model was used here rather than a CIN-based model since the SIL-framework has been used more extensively for HIV-infected populations in LMICs [12, 13, 23, 24]. Studies that chose to use a CIN-based model or an non-HIV-infected population also found VIA and/or Pap screening to be cost-effective methods of screening [44, 49–51]. We do not expect that our results would change if we had chosen to use a CIN-based model. Our results did show that if patients could receive HPV screening and treatment in a single visit (i.e., same day see-and-treat), HPV would be projected to be less expensive than VIA with equivalent or better health outcomes. Although single visit screening may be advantageous in some respects, it could lead to longer patient wait times on that day and require logistical changes for facility procedures.

This study had several limitations. First, a Markov model does not take into account disease history, such as prior lesions or changes in CD4 over time, which may impact results. Second, all strategies considered here assume that women only receive one screening per lifetime, and do not consider periodic screenings or optimal age for screening. Third, screening implementation costs such as social mobilization or demand creation were not included, which could be a barrier for a health facility considering adding screening to their current clinic services. However, HIV treatment centers generally support an already engaged population, since many patients seek services on a monthly basis for prescription refills and checkups; therefore, costs of demand generation would likely be minimal.

This study offers a societal and clinic perspective of costs of cervical cancer screening or preventative treatment. Data from a screening trial and cost study in the same clinic setting increases the strength of our analysis. There are likely many advantages to providing screening in an HIV clinic, including decreased LTFU and availability of trained nurses. However, future studies evaluating the ideal age and timing of screen among HIV-infected women would be of particular value to decision-makers and health providers. Additionally, trials should be undertaken to better understand long-term treatment effectiveness in an HIV-infected population, particularly for preventative cryotherapy.

Our results project preventative cryotherapy to be more cost-effective than cervical cancer screening for HIV-infected women. Ethical concerns for this strategy do need to be addressed, particularly for implementation in a vulnerable population such as HIV-infected women. Among screening methods, we found VIA to be the least expensive and lead to the highest life expectancy, unless HPV screening could be reduced to a single visit, in which case it may become less expensive than VIA. As such, future health systems research should focus on decrease the number of healthcare visits required for an HPV screen. Both preventative cryotherapy and VIA may be considered for widespread implementation among HIV-infected women in low-income settings.
